# Comparative analysis of pentavalent rotavirus vaccine strains and G8 rotaviruses identified during vaccine trial in Africa

**DOI:** 10.1038/srep14658

**Published:** 2015-10-06

**Authors:** Elisabeth Heylen, Mark Zeller, Max Ciarlet, Jody Lawrence, Duncan Steele, Marc Van Ranst, Jelle Matthijnssens

**Affiliations:** 1 KU Leuven - University of Leuven, Department of Microbiology and Immunology, Laboratory for Clinical and Epidemiological virology, Rega Institute for Medical Research, Leuven, Belgium; 2 Vaccines-Clinical Research Department, Merck Research Laboratories, North Wales, PA 19454, United States of America; 3 PATH, Seattle, WA, United States of America

## Abstract

RotaTeqTM is a pentavalent rotavirus vaccine based on a bovine rotavirus genetic backbone *in vitro* reassorted with human outer capsid genes. During clinical trials of RotaTeqTM in Sub-Saharan Africa, the vaccine efficacy over a 2-year follow-up was lower against the genotypes contained in the vaccine than against the heterotypic G8P[6] and G8P[1] rotavirus strains of which the former is highly prevalent in Africa. Complete genome analyses of 43 complete rotavirus genomes collected during phase III clinical trials of RotaTeqTM in Sub-Saharan Africa, were conducted to gain insight into the high level of cross-protection afforded by RotaTeqTM against these G8 strains. Phylogenetic analysis revealed the presence of a high number of bovine rotavirus gene segments in these human G8 strains. In addition, we performed an in depth analysis on the individual amino acid level which showed that G8 rotaviruses were more similar to the RotaTeqTM vaccine than non-G8 strains. Because RotaTeqTM possesses a bovine genetic backbone, the high vaccine efficacy against G8 strains might be partially explained by the fact that all these strains contain a complete or partial bovine-like backbone. Altogether, this study supports the hypothesis that gene segments other than VP7 and VP4 play a role in vaccine-induced immunity.

Group A rotaviruses (RVAs) are a major cause of severe gastroenteritis and an important etiological agent of infantile diarrhea worldwide. RVA infections cause significant morbidity and mortality (>450,000 children per year) in infants around the world, especially in low-income countries^1^.

RVAs possess a genome of 11 double-stranded RNA segments encoding six structural (VP1–VP4, VP6 and VP7) and six non-structural (NSP1–NSP6) proteins. The two outer capsid proteins, VP7 and VP4, independently elicit antibodies capable of neutralizing RVA infectivity and induce protective immunity^2^,^3^. They also form the basis of a binary classification system, determining the G- and P- genotypes of RVA strains^4^. Worldwide, G1P[8], G2P[4], G3P[8], G4P[8], G9P[8] and G12P[8] are the most common G- and P-genotype combinations, although regional differences and yearly fluctuations exists from one season to the next^5^. A uniform, sequence-based classification system for all eleven RVA gene segments was developed and is widely used^6^. Complete genome analysis is the preferred method to study the origin and evolution of human and animal RVA strains. The vast majority of all human RVA strains belong to one of two typical RVA genotype constellations referred to as Wa-like (I1-R1-C1-M1-A1-N1-T1-E1-H1) and DS-1-like (I2-R2-C2-M2-A2-N2-T2-E2-H2). The DS-1-like genotype constellation is believed to have several gene segments that have a common origin with bovine RVA strains, which is reflected in several shared genotypes between human DS-1-like RVA strains and the typical bovine RVA genotype constellation I2-R2-C2-M2-A3/11-N2-T6-E2-H3^6^.

In 2009, the WHO recommended the global use of two oral RVA vaccines, Rotarix^™^ (GlaxoSmithKline Biologicals, Rixensart, Belgium) and RotaTeq^™^ (RV5, Merck, Whitehouse Station, NJ, USA). Rotarix^™^ contains a monovalent live attenuated human G1P[8] strain, whereas RV5 contains 5 reassortant RVA strains (Fig. 1), all possessing a bovine RVA genetic constellation with a human RVA VP7 (G1-G4) or VP4 (P[8]) gene segment^7^. The WHO recommended clinical studies in Africa and Asia where disease burden is highest and oral vaccines have been less than optimal^8^,^9^,^10^,^11^,^12^,^13^. One of these studies was a double-blind, placebo-controlled Phase III clinical trial of RV5 in Ghana, Mali and Kenya, where a large genotypic diversity of RVA strains circulated within the site communities^14^. Interestingly, the VE against severe rotavirus gastroenteritis during the 2-year period of the study was numerically lower against RVA strains with the G1-G4 or P[8] genotypes contained in the vaccine (34.0%, 95% CI 11.2–51.2) than against heterotypic G8 RVAs (87.5%, 95% CI 6.5–99.7), although this study was not designed to differentiate relative efficacy against individual genotypes^14^,^15^. Tapia and colleagues described a high prevalence of G8 RVAs during this clinical trial in Africa: 5% (n = 17), 6% (n = 9) and 23% (n = 22) in Mali, Ghana and Kenya respectively^14^. The VP7 genotype G8 is a typical bovine RVA genotype, in addition to G6 and G10, which are mainly found in combination with several different P-genotypes (P[1], P[5] and P[11])^16^. A limited number of G8 rotavirus strains have been described in other animal species such as sheep, vicuña, guanaco, goat and camel^17^,^18^,^19^,^20^. G8 has also been detected in humans, with sporadic detection of G8 outside Africa and significant rates of detection inside Africa^21^,^22^,^23^,^24^,^25^,^26^,^27^,^28^,^29^,^30^,^31^,^32^.

As both the VP7 and VP4 gene segments of wild-type G8P[6] and G8P[1] RVA strains collected during the clinical trial are fully heterotypic when compared to the G- and P-genotypes present in RV5, complete genome analyses of these G8 RVA strains might help to elucidate a biological relevance of the observed high level of cross-protection afforded by RV5 against these G8 RVA strains and might also increase our understanding on how RVA vaccines afford immunity in developing world settings.

## Results

### Genotype constellations

**Figure 1:**
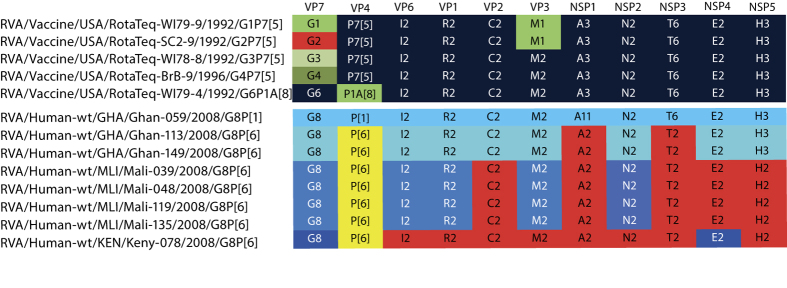
Genomic constellations of the reassortant RVA strains of RV5 and the G8 RVA strains analysed in this study. Gene segments of typical human DS-1-like RVA origin are coloured red, the human P[6] VP4 genotype is coloured yellow, and different shades of blue are used to indicate gene segments of distinct animal (most likely bovine or similar) origin.

The complete genomes of a single G8P[1] from Ghana and 7 G8P[6] RVA strains - 2 from Ghana, 4 from Mali and 1 from Kenya - were determined. Based on established genotyping criteria^33^, genotypes were assigned to each of the 11 gene segments (Fig. 1). All four Malian G8P[6] strains characterized in this study (RVA/Human-wt/MLI/Mali-039/2008/G8P[6], RVA/Human-wt/MLI/Mali-048/2008/G8P[6], RVA/Human-wt/MLI/Mali-119/2008/G8P[6] and RVA/Human-wt/MLI/Mali-135/2008/G8P[6]) possessed a DS-1-like genotype constellation. The gene segments of the two G8P[6] strains from Ghana (RVA/Human-wt/GHA/Ghan-113/2008/G8P[6] and RVA/Human-wt/GHA/Ghan-113/2008/G8P[6]) also belonged to genotype 2, except the NSP5 genotype which was classified as H3. The G8P[1] isolated in Ghana, RVA/Human-wt/GHA/Ghan-059/2008/G8P[1], possessed the G8-P[1]-I2-R2-C2-M2-A11-N2-T6-E2-H3 genotype constellation. As this combination is commonly found for bovine RVA strains, a possible bovine origin of this strain was suspected. Further phylogenetic analyses were performed in order to identify the phylogenetic relationships between the G8 RVAs characterized in this study, and to determine the most likely host origin (human or animal) of the different gene segments of these 8 G8 strains.

In order to investigate the relationship of both G8 and non-G8 DS-1-like rotavirus strains to RV5, we also sequenced the complete genomes of 35 non-G8 strains collected during the same clinical trial as the one where the G8 strains were collected. These strains all possess a complete DS-1-like background in combination with the G2P[6] (n = 17), G2P[4] (n = 11), G3P[6] (n = 6) and G1P[6] (n = 1) G- and P-genotypes. Six of these non-G8 DS-1-like strains (Ghan-013, Ghan-052, Ghan-054, Ghan-055, Mali-045 and Mali-120) were selected to include in the phylogenetic analyses, as they represented the genetic diversity of non-G8 DS-1-like strains found in this study.

### Phylogenetic analyses of VP7 and VP4

**Figure 2:**
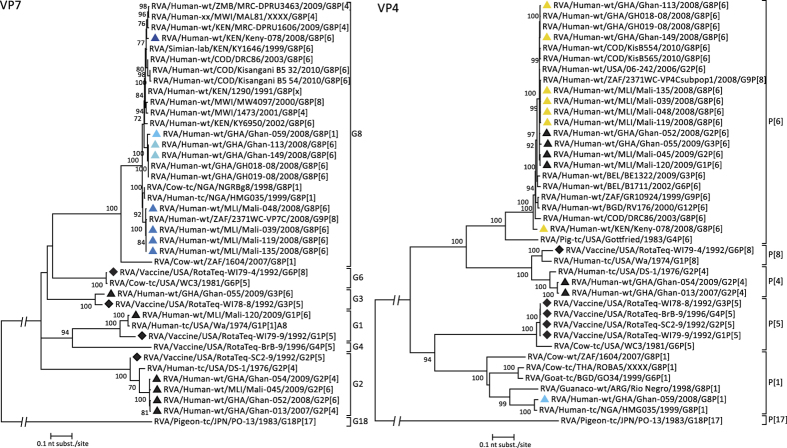
Phylogenetic dendrograms based on the nucleotide sequences of the outer capsid proteins VP4 and VP7. Bootstrap values (500 replicates) above 70 are shown. G8 RVA strains analysed in this study are indicated with a triangle (colour code according to Fig. 1), selected non-G8 strains were indicated with a black triangle, viruses present in RV5 are indicated with a diamond.

The 8 investigated G8 strains were found in three distinct clusters (95.1–100% similarity at the nt level), according to their country of isolation (Fig. 2). All four strains from Mali were completely identical on the aa level, while minor differences were found within the Ghanaian strains (98.5% aa similarity between the two G8P[6] strains and the G8P[1] strain). The 2 clusters containing strains from West-Africa (either from Ghana or from Mali) differ by only 1.8–2.8% on the aa level, while the distances between the Kenyan G8P[6] (East-Africa) and the G8P[1], Ghanaian G8P[6] and Malian G8P[6] strains were 3.7%, 2.8% and 4.0% respectively. The non-G8 strains where all homotypic compared to the G-genotypes present in RV5, clustering within the G3 (Ghan-055 and RotaTeq-WI78-8), G1 (Mali-120 and RotaTeq-WI79-9) and G2 (Ghan-054, Mali-045, Ghan-052, Ghan-013 and RotaTeq-SC2-9) genotypes.

For VP4, a clear West-East African division exists within the P[6] RVA strains, however the strain from Kenya still shared 95.6–95.9% nt identity with the P[6] strains from Ghana and Mali. In this study, all P[6] strains were more closely related to human P[6] strains, rather than to porcine P[6] strains, represented by strain RVA/Pig-tc/USA/Gottfried/1983/G4P[6] in Fig. 2. Strain Ghan-059 clusters within the P[1] genotype, and was most closely related (97.5% on the nt level) to strain RVA/Human-tc/NIG/HMG035/1999/G8P[1], a human strain believed to be of animal origin, isolated from a predominantly rural livestock-producing area in Nigeria^23^. The non-G8 strains were also heterotypic to the genotypes present in RV5, clustering within the P[6] and P[4] genotypes.

### Phylogenetic analyses of the genes forming the genetic backbone of the West-African G8P[6] RVAs

**Figure 3:**
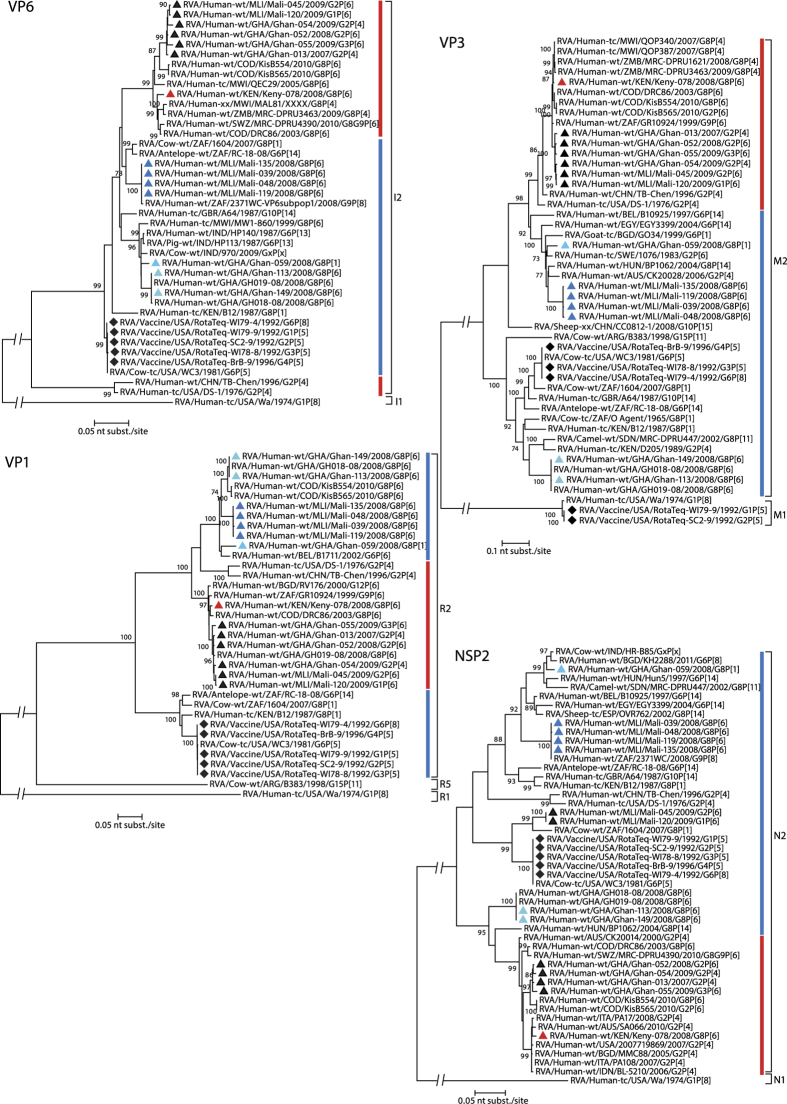
Phylogenetic dendrograms based on the nucleotide sequences of VP6, VP1, VP3 and NSP2. Bootstrap values (500 replicates) above 70 are shown. G8 RVA strains analysed in this study are indicated with a triangle (colour code according to Fig. 1), selected non-G8 strains were indicated with a black triangle, viruses present in RV5 are indicated with a diamond.

The 9 RVA gene segments encoding VP1-3, VP6, NSP1-5/6 of the six characterized West-African G8P[6] strains can be divided into 3 ‘tree-groups’, based on their clustering in the phylogenetic trees. The first ‘tree-group’ contains the VP1, VP6, VP3 and NSP2 segments of the West-African strains. Figure 3 shows that strains Ghana-113, Ghan-149, Mali-039, Mali-048, Mali-119 and Mali-135 are only distantly related to typical human DS-1-like RVA strains, which are indicated by red bars in the phylogenetic dendrograms. Instead, these genes cluster within clusters containing bovine or bovine-like human RVAs, indicated by blue bars in the phylogenetic trees. For example, for VP6, the strains from Mali are closely related to RVAs isolated from an antelope and a cow, and to the South-African strain RVA/Human-wt/ZAF/2371WC/2008/G9P[8] which has several gene segments with close similarity to artiodactyl RVAs^34^. The three Ghanaian strains are clustering together with strains RVA/Cow-wt/IND/1970/2009/GxP[x] and RVA/Pig-wt/IND/HP140/1987/G6P[13], the latter being a porcine strain with a VP6 gene of bovine origin^35^. The VP1 genes of the West-African RVA strains formed 3 distinct subclusters. The first and second subcluster contained the Ghanaian and Malian G8P[6] strains which were most closely related to RVA/Human-wt/GHA/GH018-08/2008/G8P[6], described to be a human-bovine reassortant virus^31^. The third cluster contained the G8P[1] strain, clustering most closely to strain RVA/Human-wt/BEL/B1711/2002/G6P[6], an atypical human strain which is believed to be a bovine/human reassortant strain infecting a Belgian child during a trip to Mali^36^. For VP3, the strains from Mali are most closely related to strain RVA/Human-wt/HUN/BP1062/2004/G8P[14], another atypical human strain which is the result of an interspecies transmission event^37^. During 2008, at least three different lineages of the M2 genotype circulated in Ghana, as indicated by three distinct Ghanaian clusters within the VP3 tree, showing only 83.0–88.4% nt identity. One cluster only contained non-G8 RVAs, the second cluster contained strain Ghan-059 and the third contained both strains Ghan-149 and Ghan-113. The latter two strains are very closely related to VP3 sequences of GH018-08 and RVA/Human-wt/GHA/GH019-08/2008/G8P[6], showing 99.9% nt similarity^31^. The tree of NSP2 also showed three distinct Ghanaian clusters. Two of them, which contain the Ghanaian G8 RVAs (Ghan-059 vs. Ghan-149 and Ghan-113), show 87.5% similarity on the nt level and are both subclusters of the N2 genotype not belonging to the typical human N2 cluster (indicated in red in Fig. 3). The four Malian strains are identical to 2371WC (100% nt similarity) and cluster together with unusual human P[14] RVAs indicated a shared ancestry with animal RVAs.

**Figure 4:**
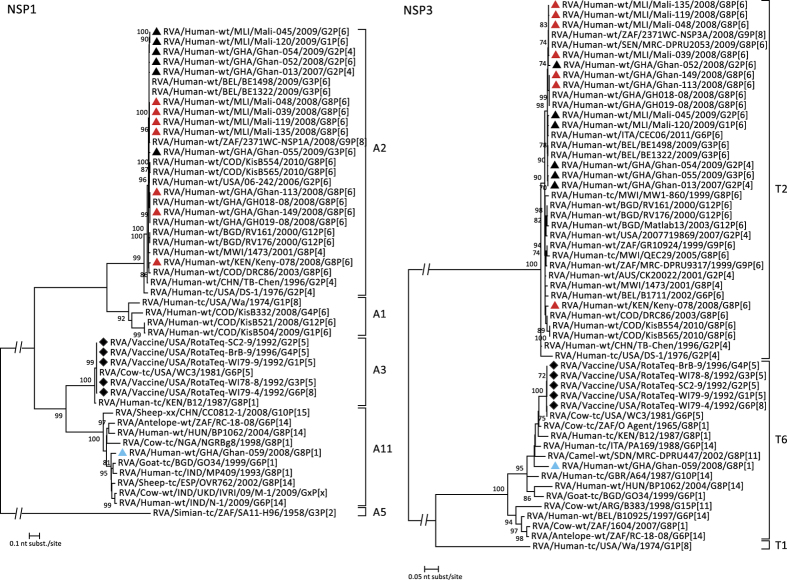
Phylogenetic dendrograms based on the nucleotide sequences of NSP1 and NSP3. Bootstrap values (500 replicates) above 70 are shown. G8 RVA strains analysed in this study are indicated with a triangle (colour code according to Fig. 1), selected non-G8 strains were indicated with a black triangle, viruses present in RV5 are indicated with a diamond.

The second ‘tree-group’ contains segments NSP1 and NSP3 (Fig. 4). Both the NSP1 and NSP3 genes of all six West-African G8P[6] RVA strains clustered closely to typical human DS-1-like RVAs isolated in countries all over the world, including Belgium (RVA/Human-wt/BEL/BE1498/2009/G3P[6] and RVA/Human-wt/BEL/BE1322/2009/G3P[6]), South-Africa (2371WC), Ghana (GH018-08 and GH019-08), Bangladesh (RVA/Human-wt/BGD/RV161/2000/G12P[6] and RVA/Human-wt/BGD/RV176/2000/G12P[6]), the Democratic Republic of the Congo (RVA/Human-wt/COD/KisB554/2010/G8P[6], RVA/Human-wt/COD/KisB565/2010/G8P[6] and RVA/Human-wt/COD/DRC86/2003/G8P[6]) and Malawi (RVA/Human-wt/MWI/1473/2001/G8P[4]).

**Figure 5:**
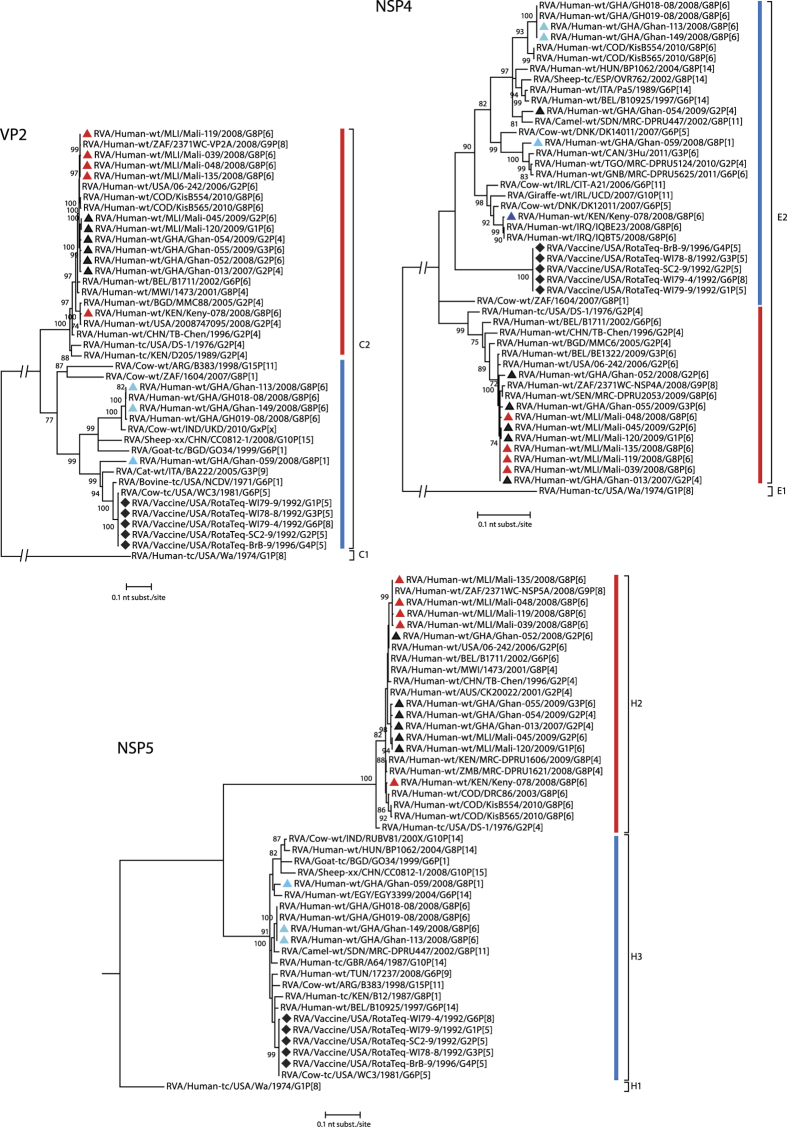
Phylogenetic dendrograms based on the nucleotide sequences of VP2, NSP4 and NSP5. Bootstrap values (500 replicates) above 70 are shown. G8 RVA strains analysed in this study are indicated with a triangle (colour code according to Fig. 1), selected non-G8 strains were indicated with a black triangle, viruses present in RV5 are indicated with a diamond.

The third ‘tree-group’ includes the remaining genes, VP2, NSP4 and NSP5. For these genes, the most likely host origin of the strains is either animal or human depending on their country of origin. Figure 5 shows that the G8P[6] strains from Ghana are more closely related to RVAs of animal origin (blue bar) than to typical human RVAs (red bar). The VP2 genes of the Ghanaian strains are divided in two clusters sharing only 85.9% nt similarity with each other and showing high similarity to the VP2 sequences of GH018-08 and GH019-08. For NSP5, the Ghanaian strains belong to genotype H3, clustering together with RVAs isolated from animals such as cow, goat and sheep. For NSP4, the three Ghanaian strains belonged to genotype E2. Both G8P[6] strains are identical and show 90.9% nt similarity to the G8P[1] strain Ghan-059. For all three genes (VP2, NSP4 and NSP5) the strains from Mali and the non-G8 strains from Ghana, formed a monophyletic cluster within the C2, E2 and H2 genotypes, clustering together with typical human RVA strains, isolated in different parts of the world.

### Strains Ghan-059 and Keny-078 show different evolutionary patterns.

Both the G8P[1] strain Ghan-059 as the East-African G8P[6] strain Keny-078, often showed another evolutionary pattern than that of the other West-African G8P[6] strains. The G8P[1] RVA strain Ghan-059 appears to possess 11 gene segments which are more closely related to bovine-like RVA strains than to typical human DS-1-like strains, including the A11 (NSP1) genotype and the T6 (NSP3) genotype, two genotypes typically found in RVAs isolated in animals which are members of the *Artiodactyla* family (cows, antelope, sheep and goat), suggesting a complete animal origin of this strain. The only G8 strain characterized from Kenya, possess the same genotype constellation as the G8P[6] strains from Mali (G8-P[6]-I2-R2-C2-M2-A2-N2-T2-E2-H2). However, the majority of its genes were more closely related to gene segments of typical human DS-1-like RVAs. The only exception was NSP4, showing a high similarity (99.6%) to G8P[6] strains isolated from Iraq which were suggested to have NSP4 genes of animal origin and to the bovine RVA strain RVA/Cow-wt/DNK/DK12011/2007/G6P[5] (97.5%)^38^,^39^.

### Genetic distances to RotaTeq^™^

The results of the phylogenetic analyses are summarized in Fig. 1, showing the most likely host origin of each segment (genes of animal origin in blue and of human origin in red); revealing that at least 4 distinct variants of G8 RVA strains co-circulated among infants in sub-Saharan Africa during 2008. The fact that all 8 G8 strains possessed a partial or complete bovine-like genetic backbone, led to the hypothesis that the observed high VE of RV5 against G8 RVAs might be explained by the fact that RV5 also possesses a bovine RVA genetic backbone. To investigate this hypothesis in more detail we compared the genetic distances (on the amino acid level) between RV5 and G8 RVA strains on the one hand, and RV5 and human non-G8 DS-1-like strains, circulating in the same area during the same period, on the other hand. Therefore, 35 human DS-1-like strains, representing the identified diversity of DS-1-like strains during the study period, were selected and completely sequenced. In total 17 G2P[6], 11 G2P[4], 6 G3P[6] and 1 G1P[6] strains with a complete DS-1-like background have been completely sequenced (table S1).

**Figure 6:**
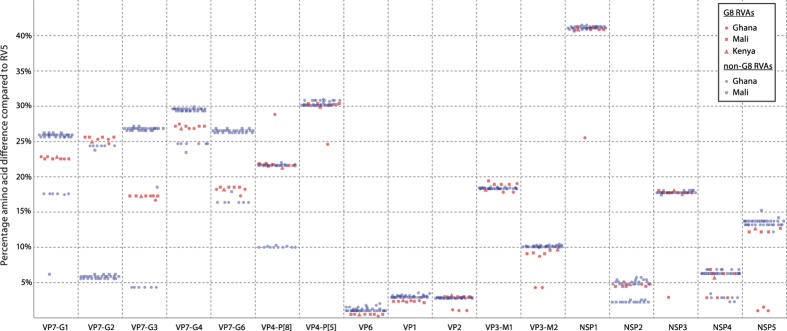
Linear plot showing the percentage of amino acid differences between gene segments of wild-type RVAs and RV5 vaccine strains. G8 strains from Ghana, Mali and Kenya are indicated by red dots, squares and triangles respectively. Non-G8 DS-1-like strains are visualized by blue dots (Ghana) or squares (Mali).

Figure 6 plots all the genetic distances between vaccine and non-vaccine strains. RV5 strain RVA/Vaccine/USA/RotaTeq-WI78-8/1992/G3P7[5] represents the X-axis of the plot; except for the genotypes not present in strain WI78-8, which are represented by strains WI79-9 (for G1 and M1), SC2-9 (for G2), BrB-9 (for G4) or WI79-4 (for P[8]). The genetic distances between G8 strains and RV5 are indicated by red symbols, while those between the non-G8 DS-1-like strains and RV5 are visualized by blue symbols. A large fraction of the symbols are plotted in the 15–45% aa difference range, as could be expected for the segments which belong to genotypes heterotypic compared to those of RV5 strains. More specifically, the VP7 (G8 instead of G1-G4 or G6), VP4 (P[6] or P[1] instead of P[8] or P[5]), VP3 (M2 instead of M1), NSP1 (A2 or A11 instead of A3), NSP3 (T2 instead of T6) and NSP5 (H2 instead of H3) genes possesses heterotypic genotypes. Some of these G8 RVA genes show smaller genetic distances to RV5 than the corresponding genes of DS-1-like RVAs (Fig. 6 and table S2). Firstly, the VP7 genotype G8 (together with the G1 and G3 genotypes) is closer related to the G6 genotype present in the vaccine than the G2 genotype often associated with DS-1-like human strains (yellow color in table S2, column VP7-G6). Secondly, the NSP1 genotype A11 (present in strains Ghan-059) showed a smaller genetic distance to the A3 genotype present in RV5 compared to the A2 genotype (74.5% aa similarity for A3 versus 58.5–59.1% for A2). Thirdly, Ghan-059 contained a T6 NSP3 gene, which shared 97.1% aa similarity with the T6 present in the vaccine. Finally, all 3 Ghanaian strains possessed the same NSP5 genotype as RV5, H3, instead of H2 present in the Malian strains and the other DS-1-like RVAs.

Focusing on the genotypes of the G8 strains, which were homotypic with the genotypes of the RV5 vaccine, minor differences between RV5/G8 RVAs and RV5/non-G8 DS-1-like RVAs were found. For example, although all VP6 genes possessed the I2 genotype and show small genetic distances to RV5, the VP6 protein of the G8 strains show only 0.3–0.5% aa differences to RV5 while the selected DS-1-like RVAs show 1.0–2.0% aa differences. The same observation can be made for VP1, showing aa differences to RV5 of 2.1–2.5% and 2.9–3.5% respectively for the G8 and non-G8 RVA strains. For VP2, VP3-M2 and NSP4 differences in genetic distances to RV5 exists between the Ghanaian and Malian G8 RVAs (as indicated by the difference in red dots and rectangles in Fig. 6) pointing out a closer relationship between the VP2, VP3 and NSP4 of RV5 and G8 RVAs isolated in Ghana than to those isolated in Mali.

Two interesting observations could be made regarding the non-G8 RVAs. First, the NSP2 of the non-G8 strains from Mali were closer related to the NSP2 of RV5, compared to all other RVAs. Second, eight Ghanaian non-G8 strains clustered together with the NSP4 of the G8 strains, which were suggested to be of animal origin.

### In-depth analysis on the individual amino acid level

**Figure 7:**
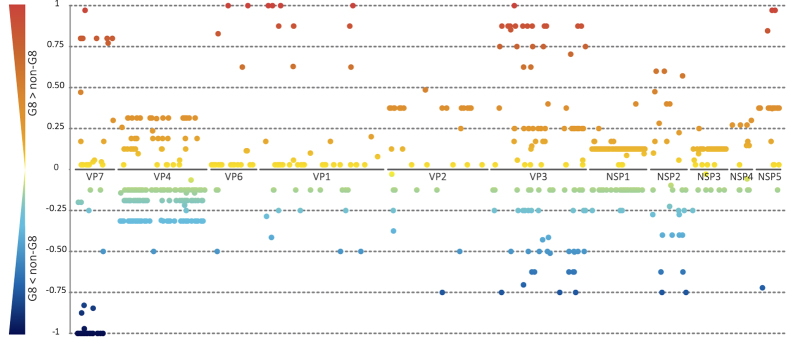
Comparison of the similarity of the G8 strains to RV5 and the non-G8 strains to RV5 per amino acid position. Each amino acid position with a score different from zero was indicated with a dot, color-coded ranging from red (1) to blue (–1). Positive similarity scores represent positions for which G8 strains were more similar to RV5 than non-G8 strains. Negative scores indicate amino acids positions were non-G8 strains are more similar to RV5 strains than G8 strains.

To evaluate if certain individual amino acid positions could contribute to the increased VE of RV5 against G8 strains, we plotted all 5771 amino acids (aa) for which data were available on a scale from –1 to 1, with a score of 1 corresponding to an aa of the G8 strains which was identical to one of those present in RV5, but was not present in the non-G8 DS-1-like RVAs (Fig. 7). In 86.33% of the aa positions the similarity score was 0 (these positions were omitted in Fig. 7), indicating no difference between the G8 and non-G8 strains versus RV5 strains. 7.82% of investigate sites showed a positive score compared to 5.85% with a negative score, indicating that more aa of the G8 RVAs were similar to RV5 than the non-G8 RVAs. Of particular interest were the aa with a score ranging between 0.5 and 1, as these positions in the G8 strains were much more similar to the RV5 strains, compared to the non-G8 RVAs. In total we identified 49 amino acids with a score ranging between 0.57 and 1. Of these, 8 aa were located in VP7, 4 in VP6, 10 in VP1, 21 within VP3, 3 in NSP2 and 3 in NSP5 (Table S3).

For VP7, none of the identified residues were located within the two structurally defined antigenic epitopes (epitopes 7-1 and 7-2)^2^. However, aa 35, was previously described to be part of the cytotoxic T cell epitope. Franco and colleagues identified aa 31–40 as an immunodominant region in the response against VP7, containing a K^b^ allele specific motif (XXXX(Y,F)XX(I,L,M,V,T)) where the one-letter code for aa is used, X represents any aa and parenthesis are used to indicate the anchor positions)^40^. This octapeptide requires a tyrosine (Y) or phenylalanine (F) at position 5, which correlated with aa 35 of VP7, and serves as a potential major histocompatibility complex class I anchor residue^41^. However, all analysed RVAs showed either a Y or F at aa 35 of VP7, keeping the K^b^ motif intact not effecting its potential role in T-cell mediated immunity. In addition to VP7, VP6, VP1 and VP3 also were described to contain multiple CTL epitopes, indicating their role in T-cell mediated immunity^42^,^43^,^44^. The non-structural proteins, NSP2 and NSP5 are known to play a role in the formation of viroplasms, which are the sites of genome replication and viral particle packaging^45^. Donker and colleagues identified a NSP2 monoclonal antibody binding epitope using phage display, modelling the antibody binding region on the NSP2 protein with a motif spanning aa 244 to aa 252^46^. However, this region does not span one of the aa identified in this study. More specifically, none of the aa residues identified in this study have been described thus fare to play a role in immunity against RVA.

## Discussion

Despite extensive research, the immunologic mechanisms and effectors responsible for protection against rotavirus after either natural infection or vaccination are still incompletely understood^47^,^48^. The recognition that multiple human rotavirus genotypes can co-circulate has long raised the critical question whether protective immunity is mainly homotypic (same G- or P-type) or rather heterotypic (different G- or P-type). The finding that heterotypic protection against severe rotavirus gastroenteritis caused by G8P[6] and G8P[1] rotavirus strains was high (87.5%, 95% CI 6.5–99.7) and statistically significant in the African clinical trial, over the 2-year follow up period of the study has indicated that high VE of RV5 in sub-Saharan Africa is possible against heterotypic genotypes^14^.

This study aimed to investigate the hypothesis that the observed high level of protection against G8 strains by RV5 could potentially be explained by the genetic backbone of the G8 strains used to estimate the VE during the clinical trial of RV5 conducted in Africa. In general, the G8 strains that drove the G8 serotype-specific VE were samples isolated in Ghana and Mali - not Kenya. Despite the high prevalence of G8 RVAs in Kenya (23%), the majority of these G8 samples were not classified as cases since they did not meet the criteria of having a Vesikari severity score of ≥11. The G8 serotype-specific analysis performed by Tapia *et al.* was based on one vaccine case and eight placebo cases (cases had to show severe rotavirus gastro-enteritis, regardless of serotype, that occurred 14 days post-dose 3), warranting caution for drawing general conclusions. More specifically, in Ghana, four G8 cases (one of which is associated with P[1]) among placebo receipts were found, three of which were completely sequenced in this study (Table S4). In Mali, four G8 cases - one in a vaccine recipient and three among placebo recipients – were detected; all four were included in this study (Table S4). The remaining G8 case used for the G8 serotype-specific analysis was reported in a placebo subject from Kenya. Unfortunately this case did not meet the selection criterion per the study protocol to make it eligible for further investigation. However, we did have the opportunity to analyse another G8 strain from Kenya which was also isolated in 2008, Keny-078. This case (Vesikari score 13) was not included by Tapia *et al.* to estimate the G8 serotype-specific VE because the G-genotype of the strain could not be determined during the initial study, but was confirmed to be G8 in a later analysis^14^.

RVA strains with at least four distinct G8 genotype constellations were identified in Ghana, Mali and Kenya with different levels of relatedness to bovine-like RV strains. Surprisingly, seven out of eight completely characterized G8 RVA strains possessed RVA genotype constellations unusual for human DS-1-like strains. More specifically, the genotype constellations of the West-African strains were shown to be largely (5 or 8 out of 11 segments) or fully of bovine or bovine-like origin. These findings suggested multiple independent interspecies transmission events followed by several reassortment events for the G8P[6] strains. However, the limited number of animal RVA sequences, especially compared to the number of human RVA sequences in Genbank, makes it difficult to determine whether the G8 strains characterized in this study are the result of a recent or more historical cross of the species boundary. More efforts are needed to sequence animal RVA strains, especially in low-income country settings. Despite the fact that no reassortment between the vaccine strains and circulating human RVA strains were found in this study, previous studies reported that reassortment between different RV5 strains and/or human RVAs is possible and can cause gastroenteritis^49^,^50^.

The fact that the G8P[1] possessed 11 gene segments of animal origin, including the P[1], T6 and A11 genotypes (typical for RVAs isolated from members of the *Artiodactyla* family), strengthens the hypothesis that this strain was the result of a direct interspecies transmission event, able to cause gastroenteritis in a human child. However the fact that this genotype was detected only in one case suggests that the G8P[1] strain was not adapted to spread among humans resulting in a dead-end infection. However, the close genetic relationship between some of the West-African G8P[6] RVA strains suggest the ability of these bovine-human reassortant RVA strains to efficiently spread from one human to another, highlighting the possibility of human and animal rotavirus strains to reassort, resulting in progeny viruses with the capability to spread in humans, pinpointing the need for continuous surveillance of rotavirus strain diversity.

The finding that the genetic backbone of the African G8 RVA strains was (partial) bovine-like led to the hypothesis that the significant protection conferred by RV5 against the heterotypic G8P[6] and G8P[1] human RVA strains could potentially be partly explained by the bovine RVA genetic backbone present in RV5. Therefore, we compared genetic distances between RV5 and G8 or non-G8 RVA strains. Minor differences between the distances of G8 and non-G8 strains were observed, often showing smaller distances between G8 RVAs and RV5 than between non-G8 RVAs and RV5. Especially the VP6, VP1 and the Ghanaian VP2, VP3 and NSP4 segments are of particular interest since these genes are more closely related to RV5 than the corresponding genes of typical human DS-1-like strains. Especially VP6 and NSP4 have been previously suggested to play a role in vaccine-induced immunity^51^,^52^,^53^,^54^,^55^. The observation that several G8 strains contained the H3 NSP5 genotype, could also be part of the explanation of the observed high VE against G8 strains.

In-depth analysis on the individual amino acid level did identify several amino acids in VP7, VP6, VP1, VP3, NSP2 and NSP5 where the G8 strains showed higher similarity to RV5 than the analysed non-G8 RVAs. Currently, none of these sites have been described to be part of rotaviral epitopes, although further studies are needed to determine if any of the identified aa belong to additional epitopes potentially present within the VP7, VP6, VP1, VP3, NSP2 or NSP5 proteins. Despite the fact that several of the observed differences are based on small numbers of samples, which does not permit robust conclusions to be made, our study may provide additional information on the possible role of other rotavirus proteins or specific amino acid residues that might play an important role in the induction of protection after natural infection or vaccination. Overall, the results of this study contribute to the understanding of why the point estimate of the vaccine efficacy against severe rotavirus gastroenteritis caused by G8 RV strains (associated with P[6] or P[1]) was higher (87.5%, 95% CI 6.5–99.7) than the efficacy against each of the individual genotypes contained in the vaccine and detected during the study: G1 (in association with any P-type), 32.3% (95% CI < 0, 55.4); G2 (in association with any P-type), 27.1% (95% CI < 0–52.2); and G3 (in association with any P-type), 62.3% (95% CI < 0–93.6). The demonstrated heterotypic protection lends further support to the hypothesis that RVA proteins other than VP7 and VP4 play a significant role in vaccine-induced immunity.

## Methods

### Study design

The samples characterized in this study were collected during a randomized, placebo-controlled phase III trial (registered with ClinicalTrials.gov, number NCT00362648 on August 8, 2006) conducted between 28 April 2007 and 31 March 2009 in three sites in Africa. More details about the study design of this clinical trial were reported previously^8^,^14^. Stool samples were collected with each diarrheal episode, if possible, and screened for the presence of RVA antigen by an enzyme immunoassay (EIA). Positive samples were G- and P-genotyped at the Merck Research Laboratories using short amplicon sequencing for VP7 and multiplex RT-PCR for VP4^56^. For this study, samples were selected by Merck/PATH based on the following criteria: (i) samples had to contribute to the per-protocol efficacy analysis, (ii) availability of sufficient stool sample (approximately 500–1500 μL of 20% raw stool suspensions), (iii) excluding EIA weakly positive samples, and (iv) permission from central and local institutional review boards and local authorities to carry out the analyses. Samples were shipped to the Rega Institute for Medical Research in Leuven, Belgium for complete genome analysis.

### Sequencing and data analysis

Double stranded RNA was extracted using the QIAamp Viral RNA mini-kit (Qiagen/Westburg, Leusden, The Netherlands) according to the manufacturer’s instructions. Subsequently reverse transcription-polymerase chain reaction (RT-PCR) was carried out at denatured RNA extracts (95 °C, 2 min followed by cooling on ice) using the Qiagen One Step RT-PCR kit (Qiagen/Westburg) with an initial RT step at 50 °C for 30 min; PCR activation was at 95 °C for 15 min, followed by 35 cycles of amplification (30 s at 94 °C, 30 s at 50 °C, and 1.5 or 6 min at 72 °C for the six shortest RVA segments and the five longest RVA segments, respectively), with a final extension of 10 min at 72 °C using the Biometra T3000 thermocycler (Biometra, Westburg BV, Netherlands). Primers used to amplify all gene segments are shown in table S5. For each sample, all 11 amplicons were pooled and sequenced on the 454 Roche GS-FLX sequencing platform (Penzberg, Germany). The sequence reads obtained from the 454 Roche GS-FLX were mapped against a VP7 and VP4 matching the previously determined G- and P-genotype of the sample completed with a Wa-like or DS-1-like background in Mira 3.4.0 or using the CLC Genomics Workbench 7.0. In cases were insufficient reads were mapped a *de novo* assembly was carried out. Sites with insufficient sequence read coverage after combining the reference mapping and the *de novo* assembly results were resequenced using the traditional Sanger sequencing method. The obtained consensus sequences were submitted to GenBank (accession numbers: Table S1) and aligned with a reference set of RVA genomes and manually edited for insertions and deletions in homopolymer regions. Using the rotavirus classification tool, RotaC (http://rotac.regatools.be), genotypes were assigned to each of the 11 gene segments^57^. In addition, to investigate the most likely host origin (human or animal) of the different gene segments phylogenetic analyses were performed. More specifically, phylogenetic trees were constructed using the maximum likelihood method with the general time reversible model in MEGA 6.0.^58^. P-distances on the amino acid (aa) level were calculated and plotted in a linear fashion.

To evaluate the similarity between G8 strains and RV5 strains on the one hand, and between non-G8 strains and RV5 strains on the other hand, a scoring system was developed. For each amino acid position the proportion of identical amino acids for G8/non-G8 and RV5 strains was determined and the difference between these proportions was used to calculate a score ranging from –1 to 1. An amino position with a score of –1 means that all the non-G8 strains were identical to RV5, while none of the G8 strains shared the same amino acid with RV5. A score of 0 means that an equal proportion of G8 strains and non-G8 strains were identical to RV5, whereas a score of 1 was defined as all G8 strains being identical to RV5, while none of the non-G8 strains were identical to RV5.

## Additional Information

**How to cite this article**: Heylen, E. *et al.* Comparative analysis of pentavalent rotavirus vaccine strains and G8 rotaviruses identified during vaccine trial in Africa. *Sci. Rep.* **5**, 14658; doi: 10.1038/srep14658 (2015).

## Supplementary Material

Supplementary Information

## References

[b1] Tate J. E. *et al.* The WHO-coordinated Global Rotavirus Surveillance Network. 2008 estimate of worldwide rotavirus-associated mortality in children younger than 5 years before the introduction of universal rotavirus vaccination programmes: a systematic review and meta-analysis. Lancet. Infect. Dis. 12, 136–141, 10.1016/S1473-3099(11)70253-5 (2012). 22030330

[b2] Aoki S. T. *et al.* Structure of rotavirus outer-layer protein VP7 bound with a neutralizing Fab. Science 324, 1444–1447, 10.1126/science.1170481 (2009). 19520960 PMC2995306

[b3] Prasad B. V., Burns J. W., Marietta E., Estes M. K. & Chiu W. Localization of VP4 neutralization sites in rotavirus by three-dimensional cryo-electron microscopy. Nature 343, 476–479, 10.1038/343476a0 (1990). 2153941

[b4] Estes M. & Greenberg H. in Fields Virology (eds Knipe D. M. *et al.* ) Ch. 45, 1347–1401 (Kluwer Health/Lippincott, Williams and Wilkins, 2013).

[b5] Banyai K. *et al.* Systematic review of regional and temporal trends in global rotavirus strain diversity in the pre rotavirus vaccine era: insights for understanding the impact of rotavirus vaccination programs. Vaccine 30 Suppl 1, A122–130, 10.1016/j.vaccine.2011.09.111 (2012). 22520121

[b6] Matthijnssens J. *et al.* Full genome-based classification of rotaviruses reveals a common origin between human Wa-Like and porcine rotavirus strains and human DS-1-like and bovine rotavirus strains. J. Virol. 82, 3204–3219 (2008). 18216098 10.1128/JVI.02257-07 PMC2268446

[b7] Matthijnssens J. *et al.* Molecular and biological characterization of the 5 human-bovine rotavirus (WC3)-based reassortant strains of the pentavalent rotavirus vaccine, RotaTeq. Virology 403, 111–127, 10.1016/j.virol.2010.04.004 (2010). 20451234

[b8] Armah G. E. *et al.* Efficacy of pentavalent rotavirus vaccine against severe rotavirus gastroenteritis in infants in developing countries in sub-Saharan Africa: a randomised, double-blind, placebo-controlled trial. Lancet 376, 606–614, 10.1016/S0140-6736(10)60889-6 (2010). 20692030

[b9] Zaman K. *et al.* Efficacy of pentavalent rotavirus vaccine against severe rotavirus gastroenteritis in infants in developing countries in Asia: a randomised, double-blind, placebo-controlled trial. Lancet 376, 615–623, 10.1016/S0140-6736(10)60755-6 (2010). 20692031

[b10] Phua K. B. *et al.* Safety and efficacy of human rotavirus vaccine during the first 2 years of life in Asian infants: randomised, double-blind, controlled study. Vaccine 27, 5936–5941, 10.1016/j.vaccine.2009.07.098 (2009). 19679216

[b11] Cunliffe N. A. *et al.* Efficacy of human rotavirus vaccine against severe gastroenteritis in Malawian children in the first two years of life: a randomized, double-blind, placebo controlled trial. Vaccine 30 Suppl 1, A36–43, 10.1016/j.vaccine.2011.09.120 (2012). 22520135 PMC3982044

[b12] Madhi S. A. *et al.* Efficacy and immunogenicity of two or three dose rotavirus-vaccine regimen in South African children over two consecutive rotavirus-seasons: a randomized, double-blind, placebo-controlled trial. Vaccine 30 Suppl 1, A44–51, 10.1016/j.vaccine.2011.08.080 (2012). 22520136

[b13] Sow S. O. *et al.* Efficacy of the oral pentavalent rotavirus vaccine in Mali. Vaccine 30 Suppl 1, A71–78, 10.1016/j.vaccine.2011.11.094 (2012). 22520140

[b14] Tapia M. D. *et al.* Secondary efficacy endpoints of the pentavalent rotavirus vaccine against gastroenteritis in sub-Saharan Africa. Vaccine 30 Suppl 1, A79–85, 10.1016/j.vaccine.2012.01.022 (2012). 22520141

[b15] Breiman R. F. *et al.* Analyses of health outcomes from the 5 sites participating in the Africa and Asia clinical efficacy trials of the oral pentavalent rotavirus Vaccine. Vaccine 30 Suppl 1, A24–29, 10.1016/j.vaccine.2011.08.124 (2012). 22520132

[b16] Papp H. *et al.* Review of group A rotavirus strains reported in swine and cattle. Vet Microbiol 165, 190–199, 10.1016/j.vetmic.2013.03.020 (2013). 23642647 PMC7117210

[b17] Matthijnssens J. *et al.* Are human P[14] rotavirus strains the result of interspecies transmissions from sheep or other ungulates that belong to the mammalian order Artiodactyla? J. Virol. 83, 2917–2929, 10.1128/JVI.02246-08 (2009). 19153225 PMC2655590

[b18] Badaracco A. *et al.* Discovery and molecular characterization of a group A rotavirus strain detected in an Argentinean vicuna (Vicugna vicugna). Vet. Microbiol. 161, 247–254, 10.1016/j.vetmic.2012.07.035 (2013). 22877519

[b19] Jere K. C. *et al.* Novel NSP1 genotype characterised in an African camel G8P[11] rotavirus strain. Infect. Genet. Evol. 21, 58–66, 10.1016/j.meegid.2013.10.002 (2014). 24184096

[b20] Parreno V., Bok K., Fernandez F. & Gomez J. Molecular characterization of the first isolation of rotavirus in guanacos (Lama guanicoe). Arch. Virol. 149, 2465–2471, 10.1007/s00705-004-0371-2 (2004). 15449134

[b21] Steele A. D. & Ivanoff B. Rotavirus strains circulating in Africa during 1996-1999: emergence of G9 strains and P[6] strains. Vaccine 21, 361–367 (2003). 12531633 10.1016/s0264-410x(02)00616-3

[b22] Cunliffe N. A. *et al.* Rotavirus G and P types in children with acute diarrhea in Blantyre, Malawi, from 1997 to 1998: predominance of novel P[6]G8 strains. J. Med. Virol. 57, 308–312 (1999). 10022804

[b23] Adah M. I., Wade A. & Taniguchi K. Molecular epidemiology of rotaviruses in Nigeria: detection of unusual strains with G2P[6] and G8P[1] specificities. J. Clin. Microbiol. 39, 3969–3975, 10.1128/JCM.39.11.3969-3975.2001 (2001). 11682516 PMC88473

[b24] Holmes J. L. *et al.* Characterization of unusual G8 rotavirus strains isolated from Egyptian children. Arch. Virol. 144, 1381–1396 (1999). 10481744 10.1007/s007050050594

[b25] Palombo E. A., Clark R. & Bishop R. F. Characterisation of a “European-like” serotype G8 human rotavirus isolated in Australia. J. Med. Virol. 60, 56–62 (2000). 10568764

[b26] Fischer T. K. *et al.* Characterization of incompletely typed rotavirus strains from Guinea-Bissau: identification of G8 and G9 types and a high frequency of mixed infections. Virology 311, 125–133 (2003). 12832210 10.1016/s0042-6822(03)00153-3

[b27] Delogu R. *et al.* Full-genome characterization of a G8P[8] rotavirus that emerged among children with diarrhea in Croatia in 2006. J. Clin. Microbiol. 51, 1583–1588, 10.1128/JCM.00396-13 (2013). 23426928 PMC3647887

[b28] Armah G. E. *et al.* Prevalence of unusual human rotavirus strains in Ghanaian children. J Med Virol 63, 67–71 (2001). 11130890

[b29] Volotao E. M. *et al.* Rotavirus surveillance in the city of Rio de Janeiro-Brazil during 2000-2004: detection of unusual strains with G8P[4] or G10P[9] specificities. J Med Virol 78, 263–272, 10.1002/jmv.20535 (2006). 16372291

[b30] Iturriza-Gomara M. *et al.* Rotavirus genotypes co-circulating in Europe between 2006 and 2009 as determined by EuroRotaNet, a pan-European collaborative strain surveillance network. Epidemiol. Infect. 139, 895–909, 10.1017/S0950268810001810 (2011). 20707941

[b31] Dennis F. E. *et al.* Identification of novel Ghanaian G8P[6] human-bovine reassortant rotavirus strain by next generation sequencing. PloS. One. 9, e100699, 10.1371/journal.pone.0100699 (2014). 24971993 PMC4074113

[b32] Istrate C. *et al.* High rate of detection of G8P[6] rotavirus in children with acute gastroenteritis in Sao Tome and Principe. Arch. Virol. 10.1007/s00705-014-2244-7 (2014). 25283609

[b33] Matthijnssens J. *et al.* Recommendations for the classification of group A rotaviruses using all 11 genomic RNA segments. Arch. Virol. 153, 1621–1629 (2008). 18604469 10.1007/s00705-008-0155-1 PMC2556306

[b34] Jere K. C., Mlera L., Page N. A., van Dijk A. A. & O’Neill H. G. Whole genome analysis of multiple rotavirus strains from a single stool specimen using sequence-independent amplification and 454(R) pyrosequencing reveals evidence of intergenotype genome segment recombination. Infect. Genet. Evol. 11, 2072–2082, 10.1016/j.meegid.2011.09.023 (2011). 22019521

[b35] Ghosh S. *et al.* Evidence for independent segregation of the VP6- and NSP4- encoding genes in porcine group A rotavirus G6P[13] strains. Arch. Virol . 152, 423–429, 10.1007/s00705-006-0848-2 (2007). 17006597

[b36] Matthijnssens J., Rahman M. & Van Ranst M. Two out of the 11 genes of an unusual human G6P[6] rotavirus isolate are of bovine origin. J. Gen. Virol. 89, 2630–2635, 10.1099/vir.0.2008/003780-0 (2008). 18796733

[b37] Banyai K. *et al.* Genetic diversity and zoonotic potential of human rotavirus strains, 2003-2006, Hungary. J. Med. Virol. 81, 362–370, 10.1002/jmv.21375 (2009). 19107981

[b38] Midgley S. E., Hjulsager C. K., Larsen L. E., Falkenhorst G. & Bottiger B. Suspected zoonotic transmission of rotavirus group A in Danish adults. Epidemiol Infect 140, 1013–1017, 10.1017/S0950268811001981 (2012). 21943834

[b39] Ahmed S. *et al.* Characterization of human rotaviruses circulating in Iraq in 2008: atypical G8 and high prevalence of P[6] strains. Infect. Genet. Evol. 16, 212–217, 10.1016/j.meegid.2012.12.003 (2013). 23340225

[b40] Franco M. A. *et al.* An immunodominant cytotoxic T cell epitope on the VP7 rotavirus protein overlaps the H2 signal peptide. J Gen Virol 74 (Pt 12), 2579–2586 (1993). 8277264 10.1099/0022-1317-74-12-2579

[b41] Falk K., Rotzschke O., Stevanovic S., Jung G. & Rammensee H. G. Allele-specific motifs revealed by sequencing of self-peptides eluted from MHC molecules. Nature 351, 290–296, 10.1038/351290a0 (1991). 1709722

[b42] Newell E. W. *et al.* Combinatorial tetramer staining and mass cytometry analysis facilitate T-cell epitope mapping and characterization. Nature biotechnology 31, 623–629, 10.1038/nbt.2593 (2013). PMC3796952 23748502

[b43] Wei J. *et al.* Identification of an HLA-A*0201-restricted cytotoxic T-lymphocyte epitope in rotavirus VP6 protein. J. Gen. Virol. 87, 3393–3396, 10.1099/vir.0.82031-0 (2006). 17030875

[b44] Wei J. *et al.* A naturally processed epitope on rotavirus VP7 glycoprotein recognized by HLA-A2.1-restricted cytotoxic CD8+ T cells. Viral immunology 22, 189–194, 10.1089/vim.2008.0091 (2009). 19435415

[b45] Eichwald C., Rodriguez J. F. & Burrone O. R. Characterization of rotavirus NSP2/NSP5 interactions and the dynamics of viroplasm formation. J. Gen. Virol. 85, 625–634 (2004). 14993647 10.1099/vir.0.19611-0

[b46] Donker N. C., Foley M., Tamvakis D. C., Bishop R. & Kirkwood C. D. Identification of an antibody-binding epitope on the rotavirus A non-structural protein NSP2 using phage display analysis. J. Gen. Virol. 92, 2374–2382, 10.1099/vir.0.032599-0 (2011). 21697352

[b47] Ward R. Mechanisms of protection against rotavirus infection and disease. Pediat.r Infect. Dis. J. 28, S57–59, 10.1097/INF.0b013e3181967c16 (2009). 19252425

[b48] Desselberger U. & Huppertz H. I. Immune responses to rotavirus infection and vaccination and associated correlates of protection. J. Infect. Dis. 203, 188–195, 10.1093/infdis/jiq031 (2011). 21288818 PMC3071058

[b49] Donato C. M. *et al.* Identification of strains of RotaTeq rotavirus vaccine in infants with gastroenteritis following routine vaccination. J. Infect. Dis. 206, 377–383, 10.1093/infdis/jis361 (2012). 22615314

[b50] Bucardo F., Rippinger C. M., Svensson L. & Patton J. T. Vaccine-derived NSP2 segment in rotaviruses from vaccinated children with gastroenteritis in Nicaragua. Infect. Genet. Evol. 12, 1282–1294, 10.1016/j.meegid.2012.03.007 (2012). 22487061 PMC3372771

[b51] Burns J. W., Siadat-Pajouh M., Krishnaney A. A. & Greenberg H. B. Protective effect of rotavirus VP6-specific IgA monoclonal antibodies that lack neutralizing activity. Science 272, 104–107 (1996). 8600516 10.1126/science.272.5258.104

[b52] Choi A. H. *et al.* Functional mapping of protective epitopes within the rotavirus VP6 protein in mice belonging to different haplotypes. Vaccine 21, 761–767 (2003). 12531356 10.1016/s0264-410x(02)00595-9

[b53] Ball J. M., Tian P., Zeng C. Q., Morris A. P. & Estes M. K. Age-dependent diarrhea induced by a rotaviral nonstructural glycoprotein. Science 272, 101–104 (1996). 8600515 10.1126/science.272.5258.101

[b54] Franco M. A., Angel J. & Greenberg H. B. Immunity and correlates of protection for rotavirus vaccines. Vaccine 24, 2718–2731, 10.1016/j.vaccine.2005.12.048 (2006). 16446014

[b55] Zhang M. *et al.* Mutations in rotavirus nonstructural glycoprotein NSP4 are associated with altered virus virulence. J. Virol. 72, 3666–3672 (1998). 9557647 10.1128/jvi.72.5.3666-3672.1998 PMC109587

[b56] DiStefano D. J. *et al.* Novel rotavirus VP7 typing assay using a one-step reverse transcriptase PCR protocol and product sequencing and utility of the assay for epidemiological studies and strain characterization, including serotype subgroup analysis. J. Clin. Microbiol 43, 5876–5880, 10.1128/JCM.43.12.5876-5880.2005 (2005). 16333070 PMC1317171

[b57] Maes P., Matthijnssens J., Rahman M. & Van Ranst M. RotaC: a web-based tool for the complete genome classification of group A rotaviruses. BMC Microbiol. 9, 238, 10.1186/1471-2180-9-238 (2009). 19930627 PMC2785824

[b58] Tamura K., Stecher G., Peterson D., Filipski A. & Kumar S. MEGA6: Molecular Evolutionary Genetics Analysis version 6.0. Mol. Biol. Evol. 30, 2725–2729, 10.1093/molbev/mst197 (2013). 24132122 PMC3840312

