# Supplementary material for: Comparative analysis of pentavalent rotavirus vaccine strains and G8 rotaviruses identified during vaccine trial in Africa
Source: Sci Rep. 2015 Oct 6;5:14658. doi: 10.1038/srep14658 (PMC4594120; doi:10.1038/srep14658)
Supplement: Supplementary Information [file srep14658-s1.pdf]

Comparative analysis of pentavalent rotavirus vaccine strains and G8 rotaviruses identified during vaccine trial in Africa

Elisabeth Heylen, Mark Zeller, Max Ciarlet, Jody Lawrence, Duncan Steele, Marc Van Ranst, Jelle Matthijnssens

Table S1. GenBank accession numbers obtained for rotavirus strains analysed in this study.

Rotavirus Strain Name	VP7	VP4	VP6	VP1	VP2	VP3	NSP1	NSP2	NSP3	NSP4	NSP5
RVA/Human-wt/GHA/Ghan-059/2008/G8P[1]	KP882472	KP882473	KP882474	KP882475	KP882476	KP882477	KP882478	KP882479	KP882480	KP882481	KP882482
RVA/Human-wt/GHA/Ghan-113/2008/G8P[6]	KP882626	KP882627	KP882628	KP882629	KP882630	KP882631	KP882632	KP882633	KP882634	KP882635	KP882636
RVA/Human-wt/GHA/Ghan-149/2008/G8P[6]	KP882681	KP882682	KP882683	KP882684	KP882685	KP882686	KP882687	KP882688	KP882689	KP882690	KP882691
RVA/Human-wt/MLI/Mali-039/2008/G8P[6]	KP882923	KP882924	KP882925	KP882926	KP882927	KP882928	KP882929	KP882930	KP882931	KP882932	KP882933
RVA/Human-wt/MLI/Mali-048/2008/G8P[6]	KP883022	KP883023	KP883024	KP883025	KP883026	KP883027	KP883028	KP883029	KP883030	KP883031	KP883032
RVA/Human-wt/MLI/Mali-119/2008/G8P[6]	KP883132	KP883133	KP883134	KP883135	KP883136	KP883137	KP883138	KP883139	KP883140	KP883141	KP883142
RVA/Human-wt/MLI/Mali-135/2008/G8P[6]	KP883176	KP883177	KP883178	KP883179	KP883180	KP883181	KP883182	KP883183	KP883184	KP883185	KP883186
RVA/Human-wt/KEN/Keny-078/2008/G8P[6]	KP882714	KP882715	KP882716	KP882717	KP882718	KP882719	KP882720	KP882721	KP882722	KP882723	KP882724
RVA/Human-wt/GHA/Ghan-052/2008/G2P[6]	KP882417	KP882418	KP882419	KP882420	KP882421	KP882422	KP882423	KP882424	KP882425	KP882426	KP882427
RVA/Human-wt/MLI/Mali-045/2009/G2P[6]	KP882989	KP882990	KP882991	KP882992	KP882993	KP882994	KP882995	KP882996	KP882997	KP882998	KP882999
RVA/Human-wt/GHA/Ghan-055/2009/G3P[6]	KP882450	KP882451	KP882452	KP882453	KP882454	KP882455	KP882456	KP882457	KP882458	KP882459	KP882460
RVA/Human-wt/GHA/Ghan-054/2009/G2P[4]	KP882439	KP882440	KP882441	KP882442	KP882443	KP882444	KP882445	KP882446	KP882447	KP882448	KP882449
RVA/Human-wt/GHA/Ghan-013/2007/G2P[4]	KP882406	KP882407	KP882408	KP882409	KP882410	KP882411	KP882412	KP882413	KP882414	KP882415	KP882416
RVA/Human-wt/MLI/Mali-120/2009/G1P[6]	KP883143	KP883144	KP883145	KP883146	KP883147	KP883148	KP883149	KP883150	KP883151	KP883152	KP883153
RVA/Human-wt/GHA/Ghan-002/2008/G2P[4]	KP882296	KP882297	KP882298	KP882299	KP882300	KP882301	KP882302	KP882303	KP882304	KP882305	KP882306
RVA/Human-wt/GHA/Ghan-004/2008/G2P[4]	KP882307	KP882308	KP882309	KP882310	KP882311	KP882312	KP882313	KP882314	KP882315	KP882316	KP882317
RVA/Human-wt/GHA/Ghan-006/2009/G3P[6]	KP882329	KP882330	KP882331	KP882332	KP882333	KP882334	KP882335	KP882336	KP882337	KP882338	KP882339
RVA/Human-wt/GHA/Ghan-007/2009/G3P[6]	KP882340	KP882341	KP882342	KP882343	KP882344	KP882345	KP882346	KP882347	KP882348	KP882349	KP882350
RVA/Human-wt/GHA/Ghan-008/2009/G2P[4]	KP882351	KP882352	KP882353	KP882354	KP882355	KP882356	KP882357	KP882358	KP882359	KP882360	KP882361
RVA/Human-wt/GHA/Ghan-009/2009/G2P[6]	KP882362	KP882363	KP882364	KP882365	KP882366	KP882367	KP882368	KP882369	KP882370	KP882371	KP882372
RVA/Human-wt/GHA/Ghan-010/2009/G2P[4]	KP882373	KP882374	KP882375	KP882376	KP882377	KP882378	KP882379	KP882380	KP882381	KP882382	KP882383
RVA/Human-wt/MLI/Mali-104/2008/G2P[6]	KP883121	KP883122	KP883123	KP883124	KP883125	KP883126	KP883127	KP883128	KP883129	KP883130	KP883131
RVA/Human-wt/GHA/Ghan-106/2009/G3P[6]	KP882582	KP882583	KP882584	KP882585	KP882586	KP882587	KP882588	KP882589	KP882590	KP882591	KP882592
RVA/Human-wt/GHA/Ghan-107/2009/G3P[6]	KP882593	KP882594	KP882595	KP882596	KP882597	KP882598	KP882599	KP882600	KP882601	KP882602	KP882603
RVA/Human-wt/GHA/Ghan-108/2009/G2P[6]	KP882604	KP882605	KP882606	KP882607	KP882608	KP882609	KP882610	KP882611	KP882612	KP882613	KP882614
RVA/Human-wt/GHA/Ghan-011/2009/G2P[4]	KP882384	KP882385	KP882386	KP882387	KP882388	KP882389	KP882390	KP882391	KP882392	KP882393	KP882394
RVA/Human-wt/GHA/Ghan-012/2009/G2P[4]	KP882395	KP882396	KP882397	KP882398	KP882399	KP882400	KP882401	KP882402	KP882403	KP882404	KP882405
RVA/Human-wt/GHA/Ghan-148/2007/G2P[4]	KP882670	KP882671	KP882672	KP882673	KP882674	KP882675	KP882676	KP882677	KP882678	KP882679	KP882680
RVA/Human-wt/MLI/Mali-028/2008/G2P[6]	KP882824	KP882825	KP882826	KP882827	KP882828	KP882829	KP882830	KP882831	KP882832	KP882833	KP882834
RVA/Human-wt/MLI/Mali-029/2008/G2P[6]	KP882835	KP882836	KP882837	KP882838	KP882839	KP882840	KP882841	KP882842	KP882843	KP882844	KP882845
RVA/Human-wt/MLI/Mali-030/2008/G2P[6]	KP882846	KP882847	KP882848	KP882849	KP882850	KP882851	KP882852	KP882853	KP882854	KP882855	KP882856
RVA/Human-wt/MLI/Mali-035/2008/G2P[6]	KP882890	KP882891	KP882892	KP882893	KP882894	KP882895	KP882896	KP882897	KP882898	KP882899	KP882900
RVA/Human-wt/MLI/Mali-036/2008/G2P[6]	KP882901	KP882902	KP882903	KP882904	KP882905	KP882906	KP882907	KP882908	KP882909	KP882910	KP882911
RVA/Human-wt/GHA/Ghan-053/2009/G2P[6]	KP882428	KP882429	KP882430	KP882431	KP882432	KP882433	KP882434	KP882435	KP882436	KP882437	KP882438
RVA/Human-wt/GHA/Ghan-056/2009/G3P[6]	KP882461	KP882462	KP882463	KP882464	KP882465	KP882466	KP882467	KP882468	KP882469	KP882470	KP882471
RVA/Human-wt/GHA/Ghan-060/2009/G2P[6]	KP882483	KP882484	KP882485	KP882486	KP882487	KP882488	KP882489	KP882490	KP882491	KP882492	KP882493
RVA/Human-wt/MLI/Mali-069/2009/G2P[6]	KP883055	KP883056	KP883057	KP883058	KP883059	KP883060	KP883061	KP883062	KP883063	KP883064	KP883065
RVA/Human-wt/MLI/Mali-070/2008/G2P[6]	KP883066	KP883067	KP883068	KP883069	KP883070	KP883071	KP883072	KP883073	KP883074	KP883075	KP883076
RVA/Human-wt/MLI/Mali-071/2008/G2P[6]	KP883077	KP883078	KP883079	KP883080	KP883081	KP883082	KP883083	KP883084	KP883085	KP883086	KP883087
RVA/Human-wt/MLI/Mali-072/2008/G2P[6]	KP883088	KP883089	KP883090	KP883091	KP883092	KP883093	KP883094	KP883095	KP883096	KP883097	KP883098
RVA/Human-wt/MLI/Mali-074/2008/G2P[6]	KP883099	KP883100	KP883101	KP883102	KP883103	KP883104	KP883105	KP883106	KP883107	KP883108	KP883109
RVA/Human-wt/GHA/Ghan-085/2008/G2P[4]	KP882516	KP882517	KP882518	KP882519	KP882520	KP882521	KP882522	KP882523	KP882524	KP882525	KP882526
RVA/Human-wt/GHA/Ghan-086/2007/G2P[4]	KP882527	KP882528	KP882529	KP882530	KP882531	KP882532	KP882533	KP882534	KP882535	KP882536	KP882537

Table S2. The percentage amino acid similarity of 43 analysed (non-G8 and G8) RVA strains compared to the different gene segments present in RV5.

		Similarity to RV5 (%)																
		VP7-G1	VP7-G2	VP7-G3	VP7-G4	VP7-G6	VP4-P8	VP4-P5	VP6	VP1	VP2	VP3-M1	VP3-M2	NSP1	NSP2	NSP3	NSP4	NSP5
non-G8 strains	RVA/Human-wt/GHA/Ghan-002/2008/G2P[4]	74.1	94.1	73.1	70.4	73.5	89.7	69.2	98.7	97.0	97.2	81.4	89.7	58.7	94.9	82.3	93.7	86.3
	RVA/Human-wt/GHA/Ghan-004/2008/G2P[4]	74.4	94.4	73.5	70.4	73.8	89.9	69.0	99.0	97.1	97.2	81.6	89.7	58.7	94.9	82.3	93.7	86.3
	RVA/Human-wt/GHA/Ghan-006/2009/G3P[6]	82.4	75.6	95.7	75.3	83.6	78.3	69.8	99.0	96.7	97.2	81.7	89.9	58.7	94.6	82.3	93.1	86.8
	RVA/Human-wt/GHA/Ghan-007/2009/G3P[6]	82.4	75.6	95.7	75.3	83.6	78.0	69.5	99.0	96.8	97.0	81.7	89.9	58.7	94.6	82.3	93.1	86.8
	RVA/Human-wt/GHA/Ghan-008/2009/G2P[4]	74.4	94.4	73.5	70.4	73.8	89.9	69.3	98.5	96.8	97.2	81.6	89.8	59.1	95.2	82.3	97.1	86.8
	RVA/Human-wt/GHA/Ghan-009/2009/G2P[6]	74.1	94.1	73.1	70.7	73.5	78.4	69.9	98.5	96.7	97.1	81.7	90.0	58.9	95.2	82.3	97.7	86.8
	RVA/Human-wt/GHA/Ghan-010/2009/G2P[4]	74.4	94.4	73.5	70.4	73.8	90.0	69.2	99.0	97.0	97.2	81.7	89.8	59.1	95.2	82.3	96.6	86.8
	RVA/Human-wt/GHA/Ghan-011/2009/G2P[4]	74.4	94.4	73.5	70.4	73.8	90.0	69.2	98.2	96.8	97.2	81.6	89.8	58.9	95.2	82.3	97.1	86.8
	RVA/Human-wt/GHA/Ghan-012/2009/G2P[4]	74.1	94.4	73.1	70.4	73.8	90.0	69.2	99.0	97.0	97.2	81.6	89.8	58.5	95.2	82.3	97.1	86.8
	RVA/Human-wt/GHA/Ghan-013/2007/G2P[4]	74.4	94.4	73.5	70.4	73.8	90.0	69.2	99.0	97.1	97.2	81.4	89.6	58.7	94.9	82.3	93.7	86.3
	RVA/Human-wt/GHA/Ghan-052/2008/G2P[6]	74.1	94.4	73.1	70.1	73.5	78.4	69.9	99.0	97.1	97.2	81.4	89.8	58.9	95.2	82.6	93.1	87.8
	RVA/Human-wt/GHA/Ghan-053/2009/G2P[6]	74.1	94.1	73.1	70.7	73.5	78.3	69.8	99.0	96.9	97.2	81.7	89.9	58.7	97.8	82.3	93.7	86.8
	RVA/Human-wt/GHA/Ghan-054/2009/G2P[4]	74.4	94.4	73.5	70.4	73.8	90.0	69.2	98.2	96.9	97.2	81.7	89.9	59.1	95.2	82.3	97.1	86.8
	RVA/Human-wt/GHA/Ghan-055/2009/G3P[6]	82.4	75.6	95.7	75.3	83.6	78.3	69.9	98.7	96.8	97.1	81.7	89.9	58.5	94.6	82.3	93.1	86.8
	RVA/Human-wt/GHA/Ghan-056/2009/G3P[6]	82.4	75.6	95.7	75.3	83.6	78.3	69.8	99.0	96.9	97.2	81.7	89.9	58.7	94.3	82.3	93.1	86.8
	RVA/Human-wt/GHA/Ghan-060/2009/G2P[6]	74.1	94.1	73.1	70.7	73.5	78.4	69.9	98.5	96.4	97.1	81.7	90.0	58.9	95.2	82.3	97.7	86.8
	RVA/Human-wt/GHA/Ghan-085/2008/G2P[4]	74.4	94.4	73.5	70.4	73.8	90.0	69.2	99.0	97.1	97.2	81.6	89.7	58.7	94.9	82.3	93.7	86.3
	RVA/Human-wt/GHA/Ghan-086/2007/G2P[4]	74.4	94.4	73.5	70.4	73.8	90.0	69.2	99.0	97.1	97.2	81.6	89.7	58.7	94.9	81.9	93.7	86.3
	RVA/Human-wt/GHA/Ghan-106/2009/G3P[6]	82.4	75.6	95.7	75.3	83.6	78.3	69.8	98.5	96.9	97.2	81.7	89.9	58.9	94.6	82.3	93.1	86.8
	RVA/Human-wt/GHA/Ghan-107/2009/G3P[6]	82.4	75.6	95.7	75.3	83.6	78.3	69.8	99.0	96.9	97.2	81.7	89.9	58.7	94.3	82.3	93.1	86.8
	RVA/Human-wt/GHA/Ghan-108/2009/G2P[6]	74.1	94.1	73.1	70.7	73.5	78.3	69.8	98.0	96.5	97.0	81.6	90.0	58.7	95.2	82.3	97.1	86.8
	RVA/Human-wt/GHA/Ghan-148/2007/G2P[4]	74.4	94.4	73.5	70.4	73.8	90.0	69.2	99.0	97.1	97.1	81.6	89.8	59.1	94.9	82.3	93.7	86.3
	RVA/Human-wt/MLI/Mali-028/2008/G2P[6]	73.8	93.8	73.1	70.4	73.1	78.4	69.9	99.0	97.1	97.1	81.7	89.9	58.9	97.8	81.9	93.7	85.8
	RVA/Human-wt/MLI/Mali-029/2008/G2P[6]	74.1	94.1	73.1	70.7	73.5	78.4	69.9	99.0	97.1	97.1	81.7	89.9	58.9	97.8	82.3	93.7	86.3
	RVA/Human-wt/MLI/Mali-030/2008/G2P[6]	74.1	94.1	73.1	70.7	73.5	78.4	69.9	99.0	97.1	97.1	81.7	89.9	58.9	97.8	82.6	93.7	86.3
	RVA/Human-wt/MLI/Mali-035/2008/G2P[6]	74.1	94.1	73.1	70.7	73.5	78.4	69.9	99.0	97.1	97.1	81.7	89.9	58.9	97.8	82.3	93.7	86.3
	RVA/Human-wt/MLI/Mali-036/2008/G2P[6]	73.8	93.8	72.8	70.4	73.1	78.4	69.9	99.0	97.0	97.1	81.7	89.9	58.9	97.8	82.3	93.7	86.3
	RVA/Human-wt/MLI/Mali-045/2009/G2P[6]	73.8	93.8	73.1	70.4	73.1	78.4	69.9	99.0	97.1	97.1	81.7	89.9	58.9	97.8	81.9	93.7	86.3
	RVA/Human-wt/MLI/Mali-069/2009/G2P[6]	73.8	93.8	72.8	70.4	73.1	78.4	69.9	99.0	97.1	97.1	81.7	89.9	58.9	97.8	82.3	93.7	86.3
	RVA/Human-wt/MLI/Mali-070/2008/G2P[6]	74.1	94.1	73.1	70.7	73.5	78.4	69.9	99.0	97.1	97.1	81.7	89.9	58.9	97.5	82.3	93.7	86.3
	RVA/Human-wt/MLI/Mali-071/2008/G2P[6]	74.1	94.1	73.1	70.7	73.5	78.6	69.9	98.7	97.1	97.1	81.7	89.9	58.9	97.5	82.3	93.7	86.3
	RVA/Human-wt/MLI/Mali-072/2008/G2P[6]	74.1	94.1	73.1	70.7	73.5	78.4	69.9	98.7	97.1	97.1	81.7	89.9	58.9	97.8	81.9	93.7	86.3
	RVA/Human-wt/MLI/Mali-074/2008/G2P[6]	74.1	94.1	73.1	70.7	73.5	78.4	69.9	98.7	97.0	97.1	81.7	89.9	58.9	97.5	82.3	93.7	84.8
	RVA/Human-wt/MLI/Mali-104/2008/G2P[6]	74.1	94.1	73.1	70.7	73.5	78.4	69.9	99.0	97.1	97.1	81.7	89.9	58.9	97.8	82.3	93.7	86.3
	RVA/Human-wt/MLI/Mali-120/2009/G1P[6]	93.8	76.2	81.5	76.5	82.1	78.3	69.8	98.7	97.0	97.1	81.6	89.7	58.9	97.8	81.9	93.7	86.3
G8 strains	RVA/Human-wt/GHA/Ghan-059/2008/G8P[1]	77.5	75.3	83.3	72.5	82.7	71.2	75.4	99.7	97.7	99.0	81.0	91.3	74.5	94.9	97.1	97.1	98.5
	RVA/Human-wt/GHA/Ghan-113/2008/G8P[6]	77.2	74.7	82.7	73.1	81.8	78.1	69.8	99.5	97.6	98.8	82.2	95.7	58.7	95.2	82.3	97.1	99.0
	RVA/Human-wt/GHA/Ghan-149/2008/G8P[6]	77.2	74.7	82.7	73.1	81.8	78.4	69.9	99.5	97.6	99.0	82.2	95.7	58.7	95.2	82.3	97.1	99.0
	RVA/Human-wt/MLI/Mali-039/2008/G8P[6]	77.5	74.4	82.7	72.8	81.5	78.4	69.6	99.5	97.7	97.0	81.1	90.8	59.1	95.5	82.3	93.7	87.3
	RVA/Human-wt/MLI/Mali-048/2008/G8P[6]	77.5	74.4	82.7	72.8	81.5	78.3	69.6	99.5	97.7	97.0	80.6	90.4	59.3	95.5	82.3	93.1	87.8
	RVA/Human-wt/MLI/Mali-119/2008/G8P[6]	77.5	74.4	82.7	72.8	81.5	78.1	69.6	99.5	97.9	97.0	81.1	90.9	59.1	95.5	81.9	93.7	87.8
	RVA/Human-wt/MLI/Mali-135/2008/G8P[6]	77.5	74.4	82.7	72.8	81.5	78.4	69.8	99.5	97.8	97.0	81.1	90.9	59.1	95.5	82.3	93.7	87.8
	RVA/Human-wt/KEN/Keny-078/2008/G8P[6]	77.2	75.0	82.7	73.1	81.8	78.7	70.0	99.5	97.5	96.8	81.8	90.3	59.1	95.2	81.9	94.3	87.3

Percentage AA similarity compared to RV5

low high

Table S3. Alignment of the 49 amino acid residues with a similarity score ranging between 0.5 and 1. Amino acids that are identical to those present in RV5 are indicated in grey.

	VP7								VP6				VP1										VP3																					NSP2			NSP5		
	35	42	55	75	178	272	281	319	64	154	281	330	67	104	160	177	289	293	294	795	806	823	69	88	143	161	167	199	209	222	275	282	316	347	359	363	459	468	486	703	767	798	835	48	121	284	95	135	162
RVA/Vaccine/USA/RotaTeq-WI79-9/1992/G1P[5]	Y	V	L	V	S	T	T	S	N	K	I	V	E	V	L	V	K	D	N	K	S	K	P	I	I	N	N	I	V	F	L	I	T	N	Q	S	V	F	I	I	A	I	E	I	H	V	T	E	V
RVA/Vaccine/USA/RotaTeq-SC2-9/1992/G2P[5]	Y	V	I	T	S	T	I	A	N	K	I	V	E	V	L	V	K	D	N	K	S	K	P	I	I	N	N	I	V	F	L	I	T	N	Q	S	V	F	I	I	A	I	E	I	H	V	T	E	V
RVA/Vaccine/USA/RotaTeq-WI78-8/1992/G3P[5]	Y	V	I	V	T	T	T	S	N	K	I	V	E	V	L	V	K	D	N	K	S	K	M	I	K	N	N	L	V	H	M	I	D	N	E	N	V	F	I	I	V	I	E	I	H	V	T	E	V
RVA/Vaccine/USA/RotaTeq-BrB-9/1996/G4P[5]	Y	V	I	N	T	T	T	S	N	K	I	V	E	V	L	V	K	D	N	K	S	K	M	I	K	N	N	L	V	H	M	I	D	N	E	N	V	F	I	I	V	I	E	I	H	V	T	E	V
RVA/Vaccine/USA/RotaTeq-WI79-4/1992/G6P[8]	Y	V	V	P	T	T	T	S	N	K	I	V	E	V	L	V	K	D	N	K	S	K	M	I	K	N	N	L	V	H	M	I	D	N	E	N	V	F	I	I	V	I	E	I	H	V	T	E	V
RVA/Human-wt/MLI/Mali-039/2008/G8P[6]	Y	V	I	P	T	T	T	S	N	K	I	V	E	V	L	V	K	D	N	K	S	K	M	I	K	N	N	L	V	H	M	I	D	N	E	S	V	F	I	I	V	I	E	I	H	V	T	E	V
RVA/Human-wt/MLI/Mali-048/2008/G8P[6]	Y	V	I	P	T	T	T	S	N	K	I	V	E	V	L	V	K	D	N	K	S	K	M	I	K	N	N	L	V	H	M	I	D	N	E	S	V	F	I	I	V	I	E	I	H	V	T	E	V
RVA/Human-wt/MLI/Mali-119/2008/G8P[6]	Y	V	I	P	T	T	T	S	N	K	I	V	E	V	L	V	K	D	N	K	S	K	M	I	K	N	N	L	V	H	M	I	D	N	E	S	V	F	I	I	V	I	E	I	H	V	T	E	V
RVA/Human-wt/MLI/Mali-135/2008/G8P[6]	Y	V	I	P	T	T	T	S	N	K	I	V	E	V	L	V	K	D	N	K	S	K	M	I	K	N	N	L	V	H	M	I	D	N	E	S	V	F	I	I	V	I	E	I	H	V	T	E	V
RVA/Human-wt/GHA/Ghan-113/2008/G8P[6]	Y	V	I	P	T	T	T	S	N	K	V	V	E	V	L	V	K	D	N	K	P	K	M	I	K	N	N	L	V	H	M	V	D	D	E	N	V	F	I	I	V	I	E	I	H	V	T	E	V
RVA/Human-wt/GHA/Ghan-149/2008/G8P[6]	Y	V	I	P	T	T	T	S	N	K	V	V	E	V	L	V	K	D	N	K	P	K	M	I	K	N	N	L	V	H	M	V	D	D	E	N	V	F	I	I	V	I	E	I	H	V	T	E	V
RVA/Human-wt/GHA/Ghan-059/2008/G8P[1]	Y	V	I	P	T	T	T	S	N	K	I	V	E	V	L	V	K	D	N	K	S	K	I	I	K	N	N	L	V	Y	M	I	D	N	E	D	I	F	I	I	V	I	G	I	H	V	T	E	V
RVA/Human-wt/KEN/Keny-078/2008/G8P[6]	Y	V	I	P	T	T	T	S	N	K	V	V	E	V	F	V	K	N	S	R	P	K	I	V	R	D	N	L	I	Y	I	V	N	T	R	D	I	L	M	V	I	V	G	I	H	V	A	E	V
RVA/Human-wt/GHA/Ghan-055/2009/G3P[6]	Y	V	I	L	T	T	T	S	N	R	V	I	K	M	F	I	K	N	S	R	P	R	I	V	R	D	N	V	I	Y	I	V	N	T	R	D	I	L	M	I	I	V	G	V	N	A	A	G	I
RVA/Human-wt/GHA/Ghan-006/2009/G3P[6]	Y	V	I	L	T	T	T	S	G	R	V	I	K	M	F	I	K	N	S	R	P	R	I	V	R	D	N	V	I	Y	I	V	N	T	R	D	I	L	M	I	I	V	G	V	N	A	A	G	I
RVA/Human-wt/GHA/Ghan-007/2009/G3P[6]	Y	V	I	L	T	T	T	S	G	R	V	I	K	M	F	I	K	N	S	R	P	R	I	V	R	-	-	V	I	Y	I	V	N	T	R	D	I	L	M	I	I	V	-	V	N	A	A	G	I
RVA/Human-wt/GHA/Ghan-106/2009/G3P[6]	Y	V	I	L	T	T	T	S	G	R	V	I	K	M	F	I	K	N	S	R	P	R	I	V	R	D	N	V	I	Y	I	V	N	T	R	D	I	L	M	I	I	V	G	V	N	A	A	G	I
RVA/Human-wt/GHA/Ghan-107/2009/G3P[6]	Y	V	I	L	T	T	T	S	G	R	V	I	K	M	F	I	K	N	S	R	P	R	I	V	R	D	N	V	I	Y	I	V	N	T	R	D	I	L	M	I	I	V	G	V	N	A	A	G	I
RVA/Human-wt/GHA/Ghan-056/2009/G3P[6]	Y	V	I	L	T	T	T	S	G	R	V	I	K	M	F	I	K	N	S	R	P	R	I	V	R	D	N	V	I	Y	I	V	N	T	R	D	I	L	M	I	I	V	G	V	N	A	A	G	I
RVA/Human-wt/MLI/Mali-120/2009/G1P[6]	Y	V	L	V	S	T	I	S	N	R	V	I	K	M	F	I	Q	N	S	R	P	R	I	V	R	D	S	V	I	Y	I	V	N	T	R	D	I	L	M	V	I	V	G	I	H	I	A	D	I
RVA/Human-wt/GHA/Ghan-052/2008/G2P[6]	F	A	M	S	N	S	I	T	N	R	V	I	K	M	F	I	K	N	S	R	P	R	I	V	R	D	S	V	I	Y	I	V	N	T	R	D	I	L	M	V	I	V	G	V	N	V	T	E	V
RVA/Human-wt/GHA/Ghan-053/2009/G2P[6]	F	A	M	S	N	S	V	T	G	R	V	I	K	M	F	I	Q	N	S	R	P	R	I	V	R	D	S	V	I	Y	I	V	N	T	R	D	I	L	M	V	I	V	G	I	H	I	A	D	I
RVA/Human-wt/MLI/Mali-045/2009/G2P[6]	F	A	M	S	N	S	V	T	N	R	V	I	K	M	F	I	Q	N	S	R	P	R	I	V	R	D	S	V	I	Y	I	V	N	T	R	D	I	L	M	V	I	V	G	I	H	I	A	D	I
RVA/Human-wt/MLI/Mali-104/2008/G2P[6]	F	A	M	S	N	S	V	T	G	R	V	I	K	M	F	I	Q	N	S	R	P	R	I	V	R	D	S	V	I	Y	I	V	N	T	R	D	I	L	M	V	I	V	G	I	H	I	A	D	I
RVA/Human-wt/MLI/Mali-028/2008/G2P[6]	F	A	M	S	N	S	V	T	G	R	V	I	K	M	F	I	Q	N	S	R	P	R	I	V	R	D	S	V	I	Y	I	V	N	T	R	D	I	L	M	V	I	V	G	I	H	I	A	D	I
RVA/Human-wt/MLI/Mali-029/2008/G2P[6]	F	A	M	S	N	S	V	T	G	R	V	I	K	M	F	I	Q	N	S	R	P	R	I	V	R	D	S	V	I	Y	I	V	N	T	R	D	I	L	M	V	I	V	G	I	H	I	A	D	I
RVA/Human-wt/MLI/Mali-030/2008/G2P[6]	F	A	M	S	N	S	V	T	G	R	V	I	K	M	F	I	Q	N	S	R	P	R	I	V	R	D	S	V	I	Y	I	V	N	T	R	D	I	L	M	V	I	V	G	I	H	I	A	D	I
RVA/Human-wt/MLI/Mali-035/2008/G2P[6]	F	A	M	S	N	S	V	T	G	R	V	I	K	M	F	I	Q	N	S	R	P	R	I	V	R	D	S	V	I	Y	I	V	N	T	R	D	I	L	M	V	I	V	G	I	H	I	A	D	I
RVA/Human-wt/MLI/Mali-036/2008/G2P[6]	F	A	M	S	N	S	V	T	G	R	V	I	K	M	F	I	Q	N	S	R	P	R	I	V	R	D	S	V	I	Y	I	V	N	T	R	D	I	L	M	V	I	V	G	I	H	I	A	D	I
RVA/Human-wt/MLI/Mali-069/2009/G2P[6]	F	A	M	S	N	S	V	T	G	R	V	I	K	M	F	I	Q	N	S	R	P	R	I	V	R	D	S	V	I	Y	I	V	N	T	R	D	I	L	M	V	I	V	G	I	H	I	A	D	I
RVA/Human-wt/MLI/Mali-070/2008/G2P[6]	F	A	M	S	N	S	V	T	G	R	V	I	K	M	F	I	Q	N	S	R	P	R	I	V	R	D	S	V	I	Y	I	V	N	T	R	D	I	L	M	V	I	V	G	I	H	I	A	D	I
RVA/Human-wt/MLI/Mali-071/2008/G2P[6]	F	A	M	S	N	S	V	T	G	R	V	I	K	M	F	I	Q	N	S	R	P	R	I	V	R	D	S	V	I	Y	I	V	N	T	R	D	I	L	M	V	I	V	G	I	H	I	A	D	I
RVA/Human-wt/MLI/Mali-072/2008/G2P[6]	F	A	M	S	N	S	V	T	G	R	V	I	K	M	F	I	Q	N	S	R	P	R	I	V	R	D	S	V	I	Y	I	V	N	T	R	D	I	L	M	V	I	V	G	I	H	I	A	D	I
RVA/Human-wt/MLI/Mali-074/2008/G2P[6]	F	A	M	S	N	S	V	T	G	R	V	I	K	M	F	I	Q	N	S	R	P	R	I	V	R	D	S	V	I	Y	I	V	N	T	R	D	I	L	M	V	I	V	G	I	H	I	A	D	I
RVA/Human-wt/GHA/Ghan-054/2009/G2P[4]	F	A	M	S	N	S	V	T	N	R	V	I	K	M	F	I	Q	N	S	R	P	R	I	V	R	D	S	V	I	Y	I	V	N	T	R	D	I	L	M	V	I	V	G	V	N	V	A	D	I
RVA/Human-wt/GHA/Ghan-013/2007/G2P[4]	F	A	M	S	N	S	V	T	N	R	V	I	K	M	F	I	K	N	S	R	P	R	I	V	R	D	S	V	I	Y	I	V	N	T	R	D	I	L	M	V	I	V	G	V	N	V	A	D	I
RVA/Human-wt/GHA/Ghan-002/2008/G2P[4]	F	A	M	S	N	S	V	T	G	R	V	I	K	M	F	I	K	N	S	R	P	R	I	V	R	D	S	V	I	Y	I	V	N	T	R	D	I	L	M	V	I	V	G	V	N	V	A	D	I
RVA/Human-wt/GHA/Ghan-004/2008/G2P[4]	F	A	M	S	N	S	V	T	G	R	V	I	K	M	F	I	K	N	S	R	P	R	I	V	R	D	S	V	I	Y	I	V	N	T	R	D	I	L	M	V	I	V	G	V	N	V	A	D	I
RVA/Human-wt/GHA/Ghan-008/2009/G2P[4]	F	A	M	S	N	S	V	T	G	R	V	I	K	M	F	I	Q	N	S	R	P	R	I	V	R	D	S	V	I	Y	I	V	N	T	R	D	I	L	M	V	I	V	G	V	N	V	A	D	I
RVA/Human-wt/GHA/Ghan-009/2009/G2P[6]	F	A	M	S	N	S	V	T	G	R	V	I	K	M	F	I	Q	N	S	R	P	R	I	V	R	D	S	V	I	Y	I	V	N	T	R	D	I	L	M	V	I	V	G	V	N	V	A	D	I
RVA/Human-wt/GHA/Ghan-010/2009/G2P[4]	F	A	M	S	N	S	V	T	G	R	V	I	K	M	F	I	Q	N	S	R	P	R	I	V	R	D	S	V	I	Y	I	V	N	T	R	D	I	L	M	V	I	V	G	V	N	V	A	D	I
RVA/Human-wt/GHA/Ghan-108/2009/G2P[6]	F	A	M	S	N	S	V	T	G	R	V	I	K	M	F	I	Q	N	S	R	P	R	I	V	R	D	S	V	I	Y	I	V	N	T	R	D	I	L	M	V	I	V	G	V	N	V	A	D	I
RVA/Human-wt/GHA/Ghan-011/2009/G2P[4]	F	A	M	S	N	S	V	T	G	R	V	I	K	M	F	I	Q	N	S	R	P	R	I	V	R	D	S	V	I	Y	I	V	N	T	R	D	I	L	M	V	I	V	G	V	N	V	A	D	I
RVA/Human-wt/GHA/Ghan-012/2009/G2P[4]	F	A	M	S	N	S	V	T	G	R	V	I	K	M	F	I	Q	N	S	R	P	R	I	V	R	D	S	V	I	Y	I	V	N	T	R	D	I	L	M	V	I	V	G	V	N	V	A	D	I
RVA/Human-wt/GHA/Ghan-060/2009/G2P[6]	F	A	M	S	N	S	V	T	G	R	V	I	K	M	F	I	Q	N	S	R	P	R	I	V	R	D	S	V	I	Y	I	V	N	T	R	D	I	L	M	V	I	V	G	V	N	V	A	D	I
RVA/Human-wt/GHA/Ghan-148/2007/G2P[4]	F	A	M	S	N	S	V	T	G	R	V	I	K	M	F	I	K	N	S	R	P	R	I	V	R	D	S	V	I	Y	I	V	N	T	R	D	I	L	M	V	I	V	G	V	N	V	A	D	I
RVA/Human-wt/GHA/Ghan-085/2008/G2P[4]	F	A	M	S	N	S	V	T	G	R	V	I	K	M	F	I	K	N	S	R	P	R	I	V	R	D	S	V	I	Y	I	V	N	T	R	D	I	L	M	V	I	V	G	V	N	V	A	D	I
RVA/Human-wt/GHA/Ghan-086/2007/G2P[4]	F	A	M	S	N	S	V	T	G	R	V	I	K	M	F	I	K	N	S	R	P	R	I	V	R	D	S	V	I	Y	I	V	N	T	R	D	I	L	M	V	I	V	G	V	N	V	A	D	I

Table S4. Details of the G8 strains completely sequenced in this study.

Sample ID	Site	Group	Sample year	VP7	VP4	Vesikari score	Case*	Genetic Background	G8 serotype-specific VE
Ghan-059	Ghana	Placebo	2008	G8	P[1]	12	Yes	I2-R2-C2-M2-A11-N2-T6-E2-H3	Yes
Ghan-113	Ghana	Placebo	2008	G8	P[6]	12	Yes	I2-R2-C2-M2-A2-N2-T2-E2-H3	Yes
Ghan-149	Ghana	Placebo	2008	G8	P[6]	17	Yes	I2-R2-C2-M2-A2-N2-T2-E2-H3	Yes
Mali-039	Mali	Placebo	2008	G8	P[6]	14	Yes	I2-R2-C2-M2-A2-N2-T2-E2-H2	Yes
Mali-048	Mali	Placebo	2008	G8	P[6]	11	Yes	I2-R2-C2-M2-A2-N2-T2-E2-H2	Yes
Mali-119	Mali	Vaccine	2008	G8	P[6]	14	Yes	I2-R2-C2-M2-A2-N2-T2-E2-H2	Yes
Mali-135	Mali	Placebo	2008	G8	P[6]	11	Yes	I2-R2-C2-M2-A2-N2-T2-E2-H2	Yes
Keny-078	Kenya	Placebo	2008	G8	P[6]	13	Yes	I2-R2-C2-M2-A2-N2-T2-E2-H2	No

*Cases were subjects with severe RVGE (as measured by Vesikari score ≥11), regardless of rotavirus serotype, occuring 14 days or more post-dose 3 of vaccine or placebo

Table S5. Primers used to amplify the VP1, VP2, VP3, VP4, VP6, VP7, NSP1, NSP2, NSP4 and NSP5 gene segments described in this study.

Gene segment	Primer name	(Degenerate) primer sequences
VP1	GEN_VP1Fb *	5′-GGC TAT TAA AGC T**R**T ACA ATG GGG AAG-3'
	GEN_VP1Rb *	5′-GGT CAC ATC TAA GCG **Y**TC TAA TCT TG -3'
VP2	GEN_VP2Fc *	5′-GGC TAT TAA AGG **Y**TC AAT GGC GTA CAG-3'
	GEN_VP2_Rbc *	5′-GTC ATA TCT CCA CA**R** TGG GGT TGG -3'
VP3	GEN_VP3Fe *	5′-GGC T**W**T TAA AGC A**R**T ATT AGT AGT G-3′
	GEN_VP3_2584R	5'- TGA CYA GTG TGT TAA GTT TYT AGC -3'
VP4	VP4-1-17F	5′-GGCTATAAAATGGCTTCGC-3′
	GEN_VP4_P8_2328R-RC	5'- CAT TGT AGA ATT A**RY** TGT TCA ATT CTA TTC C -3'
	GEN_VP4_P4_2355R-RC	5'- ACA TCC T**SR** ATG ACA TTC TCA C -3'
	GEN_VP4_P6_2359R	5'- GGT CAC ATC CTC TAT AGA GCT CTC -3'
	GEN_VP4_P1_2345R-RC	5′- GTT GCT **Y**A**Y** AAG CGA CAT TGC-3′
VP6	GEN_VP6F *	5′-GGC TTT **W**AA ACG AAG TCT TC -3
	GEN_VP6R	5′-GGT CAC ATC CTC TCA CT -3′
VP7	BEG9	5′-GGCTTTAAAAGAGAGAATTTCCGTCTGG-3′
	END9	5′-GGTCACATCATACAATTCTAATCTAAG-3′
NSP1	GEN_NSP1F	5′-GGC TTT TTT TTA TGA AAA GTC TTG -3′
	GEN_NSP1R	5'-GGT CAC ATT TTA TGC TGC C -3′
NSP2	GEN_NSP2F	5′- GGC TTT TAA AGC GTC TCA G -3′
	GEN_NSP2R	5′- GGT CAC ATA AGC GCT TTC -3′
NSP3	GEN_NSP3F	5′- GGC TTT TAA TGC TTT TCA GTG -3′
	GEN_NSP3R	5′- ACA TAA CGC CCC TAT AGC -3′
NSP4	GEN_NSP4F	5′- GGC TTT TAA AAG TTC TGT TCC -3′
	GEN_NSP4R *	5′- GG**W** **Y**AC **RY**T AAG ACC **R**TT CC -3′
NSP5	GEN_NSP5F	5′-GGC TTT TAA AGC GCT ACA G -3′
	GEN_NSP5R	5′-GGT CAC AAA ACG GGA GT -3′

* Degenerate primers; R = A/G, W = A/T, Y = C/T
